# Pharmacy-Based Travel Health Services in the United States

**DOI:** 10.3390/pharmacy7010005

**Published:** 2018-12-27

**Authors:** Keri Hurley-Kim, Jeffery Goad, Sheila Seed, Karl M. Hess

**Affiliations:** 1 Department of Pharmacy Practice, School of Pharmacy, West Coast University, Los Angeles, CA 90004, USA; khurley@westcoastuniversity.edu; 2 Department of Pharmacy Practice, School of Pharmacy, Chapman University, Irvine, CA 92618, USA; goad@chapman.edu; 3 Department of Pharmacy Practice, Massachusetts College of Pharmacy and Health Sciences University, Worcester, MA 01608, USA; sheila.seed@mcphs.edu; 4 Department of Clinical and Administrative Sciences, School of Pharmacy and Health Sciences, Keck Graduate Institute, Claremont, CA 91711, USA

**Keywords:** pharmacy law, education, training, vaccines, community pharmacy, ambulatory care

## Abstract

The aim of this paper is to review pharmacy laws and regulations, pharmacist training, clinic considerations, and patient care outcomes regarding pharmacy-based travel health services in the United States. Pharmacists and pharmacies in the United States are highly visible and accessible to the public, and have long been regarded as a source for immunization services. As international travel continues to increase and grow in popularity in this country, there is a pressing need for expanded access to preventative health services, including routine and travel vaccinations, as well as medications for prophylaxis or self-treatment of conditions that may be acquired overseas. In the United States, the scope of pharmacy practice continues to expand and incorporate these preventable health services to varying degrees on a state-by-state level. A literature review was undertaken to identify published articles on pharmacist- or pharmacy-based travel health services or care in the United States. The results of this paper show that pharmacists can help to increase access to and awareness of the need for these services to ensure that patients remain healthy while traveling abroad, and that they do not acquire a travel-related disease while on their trip. For those pharmacists interested in starting a travel health service, considerations should be made to ensure that they have the necessary training, education, and skill set in order to provide this specialty level of care, and that their practice setting is optimally designed to facilitate the service. While there is little published work available on pharmacy or pharmacist-provided travel health services in the United States, outcomes from published studies are positive, which further supports the role of the pharmacist in this setting.

## 1. Background and Methods 

In 2017, United States citizens made over 38 million visits to overseas international destinations, representing a 9% increase from the previous year, with this trend of increased travel expected to continue. According to the United States Department of Commerce, the most commonly reported purpose of travel was for vacation (55.8%), followed by visiting friends and relatives (26.7%) [1]. Regardless of the reason for travel, there are many risks involved when traveling to international destinations, from travelers’ diarrhea to malaria to yellow fever. Unfortunately, 22 to 64% of travelers report some kind of health problem that might have otherwise been prevented with travel health services [2]. However, the main source for trip planning and preparation were the airlines (52.8%), followed by an online travel agency (33.1%), which may not be ideal sources for personalized health information, recommendations, and needed vaccines or medications [1].

The field of travel medicine can be broken down into pre- and post-travel health care. Pharmacists more commonly practice pre-travel health care or simply travel health, which focuses on preventable services prior to one’s trip abroad. There is a growing need to expand access to and increase awareness of travel health services among the United States population. However, most estimates show that only about one-third to one-half of international travelers seek any form of travel health care prior to their departure from the United States [3,4]. Reasons for this include cost, accessibility, lack of awareness, and health disparities between specific U.S. populations [4,5,6]. Pharmacists can play a vital role in patient education and disease prevention related to international travel, due to their high visibility and accessibility to the public, particularly in community pharmacy settings, as well as their training. Pharmacists, depending upon state law, can often provide all necessary vaccines, medications, supplies, and in-depth patient counseling prior to their patient’s departure. The purpose of this paper is therefore to highlight United States pharmacy laws and regulations, as well as pharmacist training, travel clinic considerations, and patient care outcomes from pharmacy-based travel health services. PubMed and the bibliography section of the Pharmacists Professional Group of the International Society of Travel Medicine were searched for published articles on pharmacist or pharmacy-based travel health services or care in the United States. Published articles from outside of the United States were not used for the purpose of this review. As a result, comparisons between the United States and other countries were not undertaken. Findings from these searches are provided below. 

## 2. Main Findings

### 2.1. Pharmacists’ Scope of Practice in Travel Health in the United States: Laws and Regulations 

In the United States, the practice of pharmacy is regulated by individual states, thus there is sometimes significant variability in the care pharmacists are able to provide from one state to another. Despite this, pharmacists are highly trusted, visible to the public, and help to improve access to a variety of health care services [1,2,3,4,5,6]. Pharmacists continue to gain legal recognition as health care providers who can help support a health care system that is short on primary care physicians, nurses, and other providers. As such, the scope of pharmacists practice in certain areas, including travel health, is expanding. 

Pharmacists in the United States have been providing immunizations and travel health care services for over 20 years [7,8]. The American Pharmacists Association (APhA) reports that more than 10,000 pharmacists have received specialized travel health training (B. Shah personal communication 18 November 2018). Historically, pharmacists have provided travel health services under protocols or collaborative practice agreements (CPA) with physicians in ambulatory care settings; however, several states and territories now allow for more independent practice [7,8,9].

The travel health services that pharmacists provide can be broken down into the provision of (1) counseling, (2) administering vaccines, (3) furnishing prescription medications, (4) ordering/interpreting laboratory tests, and (5) providing self-care medications and other supplies. Of 51 jurisdictions (U.S. States and Territories), 45 allow pharmacists to provide at least some level of travel health service beyond counseling and providing self-care medications and supplies (which are within the scope of practice for all pharmacists). This includes the administration of routine and travel health vaccines, self-treatment and secondary disease prevention medications, and prophylactic medications. Travel health services may also include ordering laboratory tests, such as titers, to assess immunity to vaccine-preventable diseases as well as G6PD deficiency testing [9].

Pharmacists in 15 jurisdictions can administer all routine vaccines independently. In 30 jurisdictions, a CPA or prescription is required. Pharmacists in eight jurisdictions can administer all travel-related vaccines independently, while a CPA or prescription is required in 36 jurisdictions. Pharmacists in 25 and 19 of the jurisdictions can furnish prescription medications and order laboratory tests under a CPA, respectively. There are also specific travel health training requirements in eight states (Alaska, Arkansas, California, Florida, New Mexico, Oregon, Rhode Island, and South Carolina) [9].

### 2.2. Notable Examples

In New Mexico, pharmacists can provide all aspects of care (administering vaccines, furnishing medications, and ordering laboratory tests) independently without collaborating with a physician. In California, pharmacists can independently provide routine immunizations and travel-related prescription medications that do not require a diagnosis, which includes chemoprophylaxis and self-treatment of travel-related conditions. A CPA, however, is still required in order to administer travel vaccines in this state. In Hawaii, pharmacists can independently administer all immunizations, including those for travel, but requires a CPA in order to furnish travel-related prescription medications. Pharmacists in these three states can also independently order laboratory tests. Finally, new laws and/or regulations are pending or were recently passed in at least six jurisdictions that will expand travel health scope of practice for pharmacists if enacted into law. Interestingly, pharmacy technicians in Idaho can now administer routine vaccines to patients, which may help facilitate pharmacist-provided patient care services in this state [9].

### 2.3. Pharmacist Training

The discipline of travel health involves a comprehensive knowledge and resource base, including infectious diseases, epidemiology, environmental, geographic, and consular matters related to travelers’ health and safety [10]. Since this field is unique, dynamic, and a rapidly growing area of practice for pharmacists, it is important to maintain a high standard of practice. The following section outlines the educational and training requirements for pharmacists wanting to provide travel health services in the United States. 

Providing comprehensive travel health services involves determining patients’ specific travel health needs, providing immunizations, furnishing necessary medications, and counseling patients on health and safety risks specific to their destination and itinerary. Pharmacists in the United States interested in providing travel health services are encouraged to first complete a comprehensive immunization training program, such as the APhA Pharmacy-based Immunization Delivery Certificate Training Program (https://www.pharmacist.com/pharmacy-based-immunization-delivery) [11]. This program is comprised of a self-study and live training seminar offering 20 h continuing education. Although a general immunization-training program such as this one does not address specific travel-related vaccines in detail, it does provide a very robust and strong foundation of knowledge, practices, decision-making skills, regulations, and techniques related to immunization delivery that is necessary in patient care and travel health. 

The successful completion of the APhA Pharmacy-Based Immunization Delivery Training program and being an authorized provider of immunizations in their state is a prerequisite to enroll in the APhA Advanced Competency Training Pharmacy-Based Travel Health Services program, which helps provide a solid foundation on which to build a travel health practice. This program offers 10 h of continuing education, and includes self-study and live seminar components that will prepare pharmacists to evaluate travel itineraries; assess health and safety risks based on travelers’ destinations, reasons for travel, and medical history; and create and communicate a plan for patients to receive the necessary medications, immunizations, counseling, and non-prescription medications and supplies for their trip (https://www.pharmacist.com/pharmacy-based-travel-health-services) [12].

The gold standard in the scope of travel health knowledge is the “Body of Knowledge” developed by the International Society of Travel Medicine (ISTM). This “Body of Knowledge” serves as the basis for the Certificate of Knowledge examination that is available through the ISTM for all travel health professionals. Those who successfully complete the exam are awarded the Certificate in Travel Health (CTH) by the ISTM, which must be renewed every 10 years by continuous professional development or retesting. The CTH is one of few credentials offered across health professions and is recognized internationally (http://www.istm.org/bodyofknowledge) [13].

### 2.4. Resources

Once initial training is complete, pharmacists should maintain a comprehensive knowledge base of travel-related issues, in order to be prepared for any itinerary that may come their way. A well-informed travel health provider must have the appropriate resources to remain up-to-date on information, such as disease outbreaks, changes in country entry requirements, and vaccine recommendations [10]. The U.S. Centers for Disease Control and Prevention (CDC) maintains a list of Travel Medicine resources (https://wwwnc.cdc.gov/travel/page/travel-medicine-references) [14]. Additional travel health clinic considerations and logistics can be found in Table 1. 

### 2.5. Pharmacist and Physician Partnership for Travel Health Clinic Protocols 

State laws may require the use of a standing order, protocol, or CPA in order to administer routine and travel-related vaccines. According to the APhA Immunization Certificate Training Program, items that should be included for any vaccine protocol include:

1. Statement of physician authorization for the pharmacist to administer vaccines
2. Qualifications of person(s) administering vaccines
3. Vaccine(s) covered in the standing order/protocol
4. Policies
5. Screening patients for indications and contraindications
6. Information to provide to patients (e.g., VIS)
7. How to administer vaccine (e.g., dose, route, anatomic location)
8. Documentation requirements
9. Communication to physician and reporting requirements
10. Emergency procedures (e.g., use of epinephrine for allergic reactions) including specific protocol

Please note that only physicians can apply to become yellow fever vaccine stamp holders, but they can designate other appropriately licensed individuals at designated yellow fever vaccine centers (http://wwwnc.cdc.gov/travel/yellow-fever-vaccination-clinics/search) to administer the yellow fever vaccine and sign the international certificate of vaccination (ICV-P) [17]. Both the physician and pharmacist need to complete the appropriate yellow fever application and submit any additional documentation for certification as required by state law. 

A travel clinic protocol would, in addition to the above for vaccination, have provisions for ordering of prescription medications and ordering laboratory tests, as well as other provisions for operation (see Table 1). It is critical for pharmacists to understand that a travel immunization clinic is not a travel health clinic, and only a comprehensive travel health clinic approach that includes consultation, medications, and immunizations should be undertaken. In California, a joint statement from the state professional organizations established the standard for what a travel health clinic should provide [18]. California has a unique practice model among other states, in that pharmacists have specific authority in the law to furnish (same outcome as “prescribe”) prescription medications without a CPA, or order from a physician for international travelers, while non-routine vaccines (e.g., yellow fever, typhoid, cholera, and Japanese encephalitis) still require a CPA [19].

## 3. Outcomes of Pharmacist-Provided Care in Travel Health

More than 300,000 pharmacists have been trained to immunize in the United States [20]. Furthermore, pharmacists in all fifty states and territories are able to provide immunizations with varying degrees of restrictions, dependent upon individual regulations [9,21]. Pharmacists have been increasingly involved with providing direct patient care services that depart from the traditional dispensing role, and providing travel health services is one such activity.

In the literature, there are a few descriptive examples of pharmacist-provided care in travel health in a variety of settings, such as supermarket chain pharmacies, independent community pharmacies, telepharmacy services, multidisciplinary outpatient clinics, and student health centers [22,23,24,25]. Travel health care should include comprehensive patient consultations, and include information on disease prevention and immunizations, malaria prophylaxis, travelers’ diarrhea, insect protection, and safe food/water precautions as mentioned in the previous section [7,22,23,24]. Depending upon the type of population that the clinic serves, more specialty services could include high altitude expeditions, wilderness survival, diving, or other forms of adventure travel. 

There are limited studies in the literature that have evaluated health outcomes in pharmacist-run travel clinics in the United States. Hess et al. evaluated over 250 patients’ acceptance of pharmacist-made recommendations for vaccines and medications at an independent community pharmacy, with an 84.7% favorable patient response and a 96% patient satisfaction rate with the appointment. The study authors also showed that there was a statistically significant increase in patient knowledge of travel-related issues (medication use, adverse effects of medications, how to use insect repellents and insecticides, and how to safely consume food and water) following patient consultation. Patient satisfaction with the service also correlated with patient acceptance of pharmacist-made recommendations. This paper also documented what is believed to be the first travel health clinic located in an independent community pharmacy in the United States run solely by community pharmacists. Details on travel health clinic operations and logistics are also provided for those interested in starting up a similar service [23].

Tran et al. evaluated over 350 patients at a supermarket pharmacy. This study evaluated health outcomes, acceptance of pharmacists’ travel health recommendations, and patient satisfaction. Patients overwhelming accepted pharmacists’ recommendations for immunizations (82%–100%). Non-pharmacologic recommendations made by the pharmacist were highly accepted, at a rate of approximately 90% with regards to drinking bottled water, safe food recommendations, and the importance of washing hands. A reported 20% of patients experienced travelers’ diarrhea while traveling; however, those that experienced diarrhea and used the medications recommended by the pharmacist saw their symptoms alleviated. Approximately 79% obtained information on the prevention of malaria and insect protection. More than 90% reported that they took the medications as directed, and none contracted malaria while traveling [26]. Both Hess et al. and Tran et al. reported high patient satisfaction rates (96% and 94%, respectively) with a pharmacist-run travel health clinic [23,26].

Durham et al. compared recommendations between trained pharmacists at a pharmacist-run travel clinic in a university student health clinic versus primary care providers (PCP) without specialized training. Of the 513 travelers reviewed, pharmacists were more likely to follow evidence-based guidelines in regards to prescribing antibiotics for travelers’ diarrhea when indicated (96% versus 50%), prescribing appropriate antimalarial medications (98% versus 81%), and ordering more vaccines for patients (mean 2.77 versus 2.31). Patients were also more likely to fill antibiotic prescriptions from the pharmacists-led clinic than from prescriptions written by their PCP (75% versus 63%) [25].

These studies show high patient satisfaction rates with pharmacist-provided travel health services, including both pharmacologic and non-pharmacologic recommendations with promising health outcomes (see Table 2 for full details). Despite these high satisfaction rates, some patients refused the pharmacist’s recommendation for various vaccines. Patient acceptance rates in Tran et al. ranged from 10% (for Japanese encephalitis) to 100% (for yellow fever) for travel-related vaccines, but routine vaccine acceptance rates ranged from 0% to 31% [26]. Hess et al. had similar acceptance rates of travel-related vaccines, ranging from 67% (for polio) to 97% (for yellow fever). Patients cited a self-perceived low risk of contracting illnesses, or were only focused on the travel-related vaccines, such as yellow fever or typhoid, and not on routine illnesses like influenza or measles, mumps, and rubella [23]. Durham et al. demonstrated that pharmacists with specialty training and CTH credentials were able to provide expert care for their patients in regards to travel health [25]. All studies demonstrated that pharmacists are integral in educating patients on how to maintain their health while traveling abroad. In an effort to decrease refusal rates and increase immunization rates, pharmacists need to be more effective in informing patients on the risks of contracting vaccine-preventable travel related infectious diseases during the pre-travel visit. More research on the impact of such education and health outcomes for patients traveling is needed. 

## 4. Conclusions

The main limitation of this review is the lack of previously published literature that describes and assesses pharmacy- and pharmacist-provided travel health services in the United States. However, this paper adds to existing literature and provides an overview of the status of travel health care in the United States, including current U.S. pharmacy laws and regulations. Furthermore, practical considerations to incorporate such services has been provided to help further expand patient access to this needed service. This, in turn, may help to increase awareness of travel-related disease risks among the traveling public, and help to drive utilization of travel health care into the pharmacy setting.

## Figures and Tables

**Table 1:** Travel health clinic considerations and logistics [15,16].

Components	Comments
Patient education material	Printed or electronic (must be current). Patient-and itinerary-specific. U.S. Centers for Disease Control and Prevention (CDC) and commercial sources have patient handouts.
Immunization	With the exception of yellow fever vaccine, most immunizations are available to order through pharmacy wholesalers or other vaccine distributors. Yellow fever vaccine is supplied directly by the manufacturer, and may only be ordered by facilities associated with an official yellow fever vaccine provider. As with basic immunization services, it is important that all necessary supplies and equipment for administration are available and easily accessible. Close attention should be paid to the storage requirements of all vaccines. See the CDC’s recommendation for proper storage and handling of all vaccines. (http://www.cdc.gov/vaccines/hcp/admin/storage/toolkit/index.html).
Provision of prescription medications	Furnishing, prescribing, initiating, and ordering medications (term varies by state) Medications recommended for international travel that the pharmacists may furnish or provide generally fall into two categories: ⚬ self-treatment ⚬ chemoprophylaxis. The CDC Yellow Book details all drugs and conditions that fall into these categories. Many travel health practices opt to use pre-populated checklist-type prescription forms, as the regimens for common travel related medications are standard. This may help to increase efficiency, consistency, and potentially reduce furnishing errors. All furnishing pharmacists in need to obtain an individual National Provider Identification (NPI).
Laboratory tests	State law dictates how or if pharmacists can order tests. Antibody titers: ⚬ Hepatitis A and B ⚬ Varicella Zoster Virus (VZV) ⚬ Measles, Mumps, and Rubella (MMR) ⚬ Rabies Glucose-6 Phosphate Dehydrogenase (G6PD) deficiency for primaquine and tafenoquine.
Supplies	Best to stock an adequate supply for sale, but can create patient handouts of supplies to obtain elsewhere. Over-the-counter supplies. Non-medication supplies.
Workflow	Perform the risk assessment based on the patient’s travel health history. Prepare patient specific education documents and recommendations. Provide the travel consultation. Provide appropriate immunizations and documentation.
Staffing	Marketing, patient scheduling and reminders, and vaccine/prescription input and billing can be delegated to a pharmacy technician, clerk, or intern pharmacist (i.e., a student pharmacist in training). In an ambulatory care setting, nurses may also be used to perform clerical responsibilities and administer vaccinations. A student pharmacist may also assist in the preparation of the consultation documents and recommendations, and preparation and administration of vaccinations if appropriately trained and supervised.
Space	The space used for existing services, such as routine immunizations, is usually appropriate for providing travel health services. A private clinic room is ideal, but not required, as patients may feel more comfortable discussing medical history and receiving immunizations in an enclosed area.
Scheduling of Patients	Appointment (preferred), but can do walk-in. Schedule for a minimum of 30 minutes, depending upon the complexity of the visit. Ask patients to make appointments four to six weeks before departure. Focused travel clinic visits rather than integrating with other services. Consider group consults for families traveling together or groups with the same itinerary.
Documentation	Documentation can be print or electronic—states that require immunization registry documentation will need electronic transmission. A patient progress note that fully documents the clinical assessment and travel medication plan. A patient medication record for each medication provided to the patient by the pharmacist. Documentation of the administration of vaccines (vaccine name, lot number, expiration date, anatomical site vaccine administered, initials of pharmacist, date vaccine given, date of Vaccine Information Statement (VIS) Documentation of yellow fever vaccination on the International Certificate of Vaccination or Prophylaxis (ICV-P) form with associated official stamp from the state health department when yellow fever vaccine is administered. Documenting refrigerator and freezer temperatures at least twice a day following CDC recommendations. This is also a requirement of being a yellow fever vaccine provider.

**Table 2:** Outcomes from pharmacist-based travel health services.

**Authors**	Hess et al. [23]	Durham et al. [25]	Tran et al. [26]
**Methods**	Retrospective database review of patient records and prospective patient satisfaction survey (4-point Likert scale) of patients seen at a pharmacist-run travel health clinic in an independent pharmacy.	Retrospective chart review comparing patients seen by a clinical pharmacist in a pharmacist-run travel clinic or a primary care provider (PCP) for international travel at a student health center at a university.	Retrospective cross-sectional study conducted in supermarket pharmacy. Telephone interview (75-question survey) for those patients that received a travel consultation.
**Number of Eligible Subjects/Completed Study**	283/82	513/172 (PCP) and 341 (Pharmacist)	356/103
**Demographics**	Database review: Average age: 47 years Female: 59% Survey: Average age: 52 years Female: 69% Completed college: 39%	Average age (18-25 years): 74% Females: 64%	Average age: 44 years Male: 47% Completed college: 75%
**Objectives**	Evaluate effectiveness of a pharmacist-run travel clinic through analysis of patient acceptance and refusal rates of recommendations, changes in understanding of travel-related issues and patient satisfaction with services. Explore factors that influence recommendations made with the patient’s understanding of travel-related issues and patient satisfaction.	Compare and assess travel-related vaccine and medication recommendations between primary care providers and clinical pharmacists, with a specialty in pre-travel health. Compare compliance of medications and vaccinations recommended in each group.	Evaluate health outcomes and acceptance rates of travel health recommendations made by a pharmacist, and assess patient satisfaction rates with travel health-related services.
**Results**	Acceptance of pharmacist recommended vaccines/medications: Total acceptance rate: 85% Antimalarials: 94% Yellow fever: 97% Polio: 66% Meningococcal: 71% Typhoid: 77% Hepatitis A: 79% Reasons for refusal: Perceived low-risk of illness: 52% Only wanted yellow fever vaccine: 14% Cost: 14% Do not like receiving vaccines or taking medications: 7% Not confident in recommendation made: 3% Concerned about possible adverse effects: 3% Changes in patient understanding: Before and after (mean, *p*-value): How to use travel meds correctly: 2.51 vs. 3.82, *p* < 0.05 Possible side effects of travel medications: 2.4 vs. 3.75, *p* < 0.05 How to use insect repellents correctly: 2.95 vs. 3.73, *p* < 0.05 How to safely consume food and water: 3.22 vs. 3.82, *p* < 0.05 Overall patient satisfaction*: 3.73 (mean)	Pharmacist vs. PCP Ordered antibiotics when indicated: 96% vs. 50%, *p* < 0.0001 Received antibiotics: 74.62% vs. 62.96%, *p* = 0.0359 Ordered antimalarial when indicated: 97.78% vs. 81.02%, *p* < 0.001 Received antimalarials: 81.48% vs. 86.36%, *p* = 0.2657 Ordered vaccines when indicated (mean number of vaccines): 2.78 vs. 2.06, *p* < 0.0001 Received vaccines (mean number of vaccines): 2.38 vs. 1.95, *p* = 0.0039	Acceptance of immunization recommendations: Hepatitis A: 67% Hepatitis B: 19% Influenza: 13% Japanese encephalitis: 10% Meningococcal: 18% Measles, mumps, rubella: 31% Polio: 79% Typhoid: 82% Yellow fever: 100% Accepting pharmacist travel health recommendations: Prevention of sunburn: ⚬ Applied sunscreen: 87% Prevention of travelers’ diarrhea: ⚬ Washed hands: 89% ⚬ Drank bottled water: 89% ⚬ Ate well-cooked food: 82% Insect protection: ⚬ Applied insect repellent: 61% ⚬ Wore protective clothing: 61% ⚬ Obtained antimalarial medications: 79% (of which 92% completed therapy) Prevention of altitude sickness: ⚬ Ascended slowly: 75% ⚬ Ate high-carbohydrate diet: 17% Health Outcomes: 20% reported adverse effects with immunizations 5% reported a sunburn during their trip 20% reported travelers’ diarrhea during trip 26% reported mosquito bites during their trip 0% reported contracting malaria 0% reported altitude sickness Overall patient satisfaction**: 4.75 (mean)	
**Limitations**	Low response rate (29%), potential for recall bias since the survey was completed up to 1 year after clinic visit.	Not generalizable to general population, since the study only consisted of college-aged students. Could not control for differences in postgraduate training of the PCP’s.	Low response rate (29%), the survey was delivered by telephone, and did not include questions on why the patient did not accept or follow the recommendations completely during travel.

PCP: Primary Care Provider; * 4-point Likert scale; ** 5-point Likert scale.

## References

[1] ITA Office of Travel and Tourism Industries US Outbound Travel by World Regions. http://tinet.ita.doc.gov/outreachpages/outbound.general_information.outbound_overview.asp.

[2] Steffen R., Keystone J.S. (2004). Epidemiology: Morbidity and Mortality in Travelers. Travel Medicine.

[3] LaRocque R.C., Rao S.R., Tsibris A., Lawton T., Anita Barry M., Marano N., Brunette G., Yanni E., Ryan E.T. (2010). Pre-travel health advice-seeking behavior among US international travelers departing from Boston Logan International Airport. J. Travel Med..

[4] Hamer D.H., Conner B.A. (2004). Travel health knowledge, attitudes and practices among United States travelers. J. Travel Med..

[5] Leonard L., Van Landingham M. (2001). Adherence to travel health guidelines: The experience of Nigerian immigrants in Houston, Texas. J. Immigr. Health.

[6] Angell S.Y., Cetron M.S. (2005). Health disparities among travelers visiting friends and relatives abroad. Ann. Intern. Med..

[7] Seed S.M., Spooner L.M., O’Connor K., Abraham G.M. (2011). A Multidisciplinary approach in travel medicine: The pharmacist perspective. J. Travel Med..

[8] Jackson A.B., Humphries T.L., Nelson K.M., Helling D.K. (2004). Clinical pharmacy travel medicine services: A new frontier. Ann. Pharmacother..

[9] Hurley-Kim K., Snead R., Hess K.M. (2018). Pharmacists’ scope of practice in travel health: A review of state laws and regulations. J. Am. Pharm. Assoc..

[10] Hill D.R., Ericsson C.D., Pearson R.D., Keystone J.S., Freedman D.O., Kozarsky P.E., DuPont H.L., Bia F.J., Fischer P.R., Ryan E.T. (2006). The practice of travel medicine: Guidelines by the Infectious Diseases Society of America. Clin. Infect. Dis..

[11] American Pharmacists Association Pharmacy Based Immunization Delivery. https://www.pharmacist.com/pharmacy-based-immunization-delivery.

[12] American Pharmacists Association Pharmacy Based Travel Health Services. https://www.pharmacist.com/pharmacy-based-travel-health-services.

[13] International Society of Travel Medicine ISTM Certificate of Knowledge. http://www.istm.org/bodyofknowledge.

[14] Centers for Disease Control and Prevention Travel Medicine References: Books, Journals Articles, and Websites. https://wwwnc.cdc.gov/travel/page/travel-medicine-references.

[15] Gregorian T., Bach A., Hess K., Goad J., Mirzaian E. (2017). Implementing Pharmacy-Based Travel Health Services: Insight and Guidance from Frontline Practitioners. Calif. Pharm. J..

[16] Centers for Disease Control and Prevention Vaccine Storage and Handling Toolkit. http://www.cdc.gov/vaccines/hcp/admin/storage/toolkit/index.html.

[17] Centers for Disease Control and Prevention. http://wwwnc.cdc.gov/travel/yellow-fever-vaccination-clinics/search.

[18] Goad J., Dudas V., Gregorian T., McCabe J., Hess K., Soleimanpou S. (2016). Practice of Travel Health for Pharmacists. Joint California Pharmacist Association and California Society of Health-Systems Pharmacists Sub-Commit-Tee on SB493 Travel Medicine Provision. https://tinyurl.com/travelhealthCA.

[19] Pharmacists Furnishing Travel Medications, Division 17 of Title 16 of the California Code of Regulations. https://www.pharmacy.ca.gov/laws_regs/1746_5_oa.pdf.

[20] American Pharmacists Association (APhA) APhA Honors 2018 Immunization Champions. https://www.pharmacist.com/article/apha-honors-2018-immunization-champions.

[21] Hogue M.D., Grabenstein J.D., Foster S.L., Rotholz M.C. (2006). Pharmacist involvement with immunizations: A decade of professional advancement. J. Am. Pharm. Asscoc..

[22] Gatewood S.B., Stanley D.D., Goode J.V. (2009). Implementation of a comprehensive pretravel health program in a supermarket chain pharmacy. J. Am. Pharm. Assoc..

[23] Hess K.M., Dai C.W., Garner B., Law A.V. (2010). Measuring Outcomes of a Pharmacist-Run Travel Health Clinic Located Within an Independent Community Pharmacy. J. Am. Pharm. Assoc.

[24] Helling D.K., Nelson K.M., Ramirez J.E., Humphries T.L. (2006). Kaiser Permanente Colorado region pharmacy department: Innovative leader in pharmacy practice. J. Am. Pharm. Assoc..

[25] Durham M.J., Goad J.A., Neinstien L.S., Lou M. (2011). A comparison of pharmacist travel-health specialists’ versus primary care providers’ recommendations for travel-related medications, vaccination, and patient compliance in a college health setting. J. Travel Med..

[26] Tran D., Gatewood S., Moczygemba L.R., Stanley D.D., Goode J.V. (2015). Evaluating health outcomes following a pharmacist-provided comprehensive pretravel health clinic in a supermarket pharmacy. J. Am. Pharm. Assoc..

